# Editorial: Breakthrough in Imaging-Guided Precision Medicine in Oncology

**DOI:** 10.3389/fonc.2022.908561

**Published:** 2022-05-18

**Authors:** Ashley Shaw, Romain-David Seban, Florent L. Besson, Helena Vila-Reyes, Samy Ammari, Fatima-Zohra Mokrane, Randy Yeh, Laurent Dercle

**Affiliations:** ^1^ Department of Radiology, NewYork-Presbyterian, Columbia University Irving Medical Center, New York, NY, United States; ^2^ Department of Nuclear Medicine, Institut Curie, Paris, France; ^3^ Laboratory of Translational Imaging in Oncology, Paris Sciences et Lettres University (PSL) Research University, Institut Curie, Orsay, France; ^4^ Department of Nuclear Medicine, Université Paris-Saclay, Le Kremlin-Bicêtre, France; ^5^ Department of Medical Imaging, Institut Gustave Roussy, Villejuif, France; ^6^ Department of Radiology, Faculté de Médecine Rangueil, Université Toulouse III Paul Sabatier, Toulouse, France; ^7^ Department of Radiology, Memorial Sloan Kettering Cancer Center, New York, NY, United States

**Keywords:** radiomic, artificial intelligence, deep learning, machine learning, oncology, computed tomography, positron - emission tomography, magnetic resonance imaging

## Introduction

In the era of precision medicine in oncology, medical imaging is pivotal for a broad spectrum of indications, ranging from early detection of malignant lesions to response assessment in advanced metastatic disease. The Research Topic “*Breakthrough in imaging-guided precision medicine in oncology*” aimed to share disruptive technologies in the field of imaging-guided precision medicine in oncology. The goal was to discuss new concepts and discoveries in the field of imaging biomarkers derived from a quantitative analysis of data contained in medical images. This editorial aims to provide an at-a-glance overview of the 34 articles from 282 authors published in this guest editorial, which collectively support the concept that imaging biomarkers can be used as clinical decision tools, benefiting the outcomes of oncologic patients. Moreover, it provides an overview of research trends at the crossroads between radiology, nuclear medicine, computer science, biochemistry, pathology, and oncology.

## New Imaging Technologies for Precision Medicine

### Software: Radiomics, Machine-Learning, Deep-Learning, Artificial Intelligence

Capturing the general complexity of tumor biology has become a key challenge in precision medicine. Boosted by the recent evolution of computer science and artificial intelligence (AI) in the field of medical imaging, new powerful image-based analyses have emerged. Radiomics, a large-scale image-based approach derived from OMIC, requires sophisticated workflows consisting of image acquisition, lesion segmentation, feature extraction, and machine learning that has benefited -and could further benefit- from controlling its variation (Zhao et al.).

In this guest editorial, several authors have unraveled potential indications of radiomics for precision medicine approaches. To face the growing complexity of multidimensional quantitative data, AI appears to be a promising way to assist practitioners in the future. AI can extract and quantify key image information such as its morphological, textural, and molecular features, which can convert subjective qualitative tasks to objective quantitative analysis (Li et al.). For instance, radiomics-based imaging biomarkers, such as 18F-fluciclovine, could be utilized in the detection and management of biochemical-recurrent prostate cancer (Shaikh et al.). Radiomics-based nomogram on 18F-Fluorodeoxyglucose (FDG) positron emission tomography/computed tomography scan (PET/CT) rad-score, combined with clinicopathological factors, was proposed as a new method to personalize management of patients with non-small cell lung cancer. This approach may effectively strengthen the limitations of TNM staging methodology when evaluating lung cancer prognosis (Yang et al.). These radiomics models combining multi-omics analyses could provide a more holistic overview of tumor behavior and predictive capabilities. As an example, combining AI with ultrasound and pathological tests was utilized to tailor personalized therapies for patients with thyroid cancer (Li et al.). Additionally, radiomics-based prediction of histological subtypes was identified through the use of activation maps, which identified specific regions responsible for signature activation and was used to guide treatment decisions in non-small cell lung cancer (Vuong et al.).

There is an ongoing quest toward obtaining reproducibility and generalizable results to characterize image phenotypes (Zhao et al.). Therefore, understanding and controlling the sources of variation is necessary. This requires collaborative multidisciplinary approaches with consistent workflows (Zhao et al.). One approach to mitigate variability is by performing standardized image processing. As illustrated in (Ammari et al.), brain magnetic resonance imaging (MRI) pre-processing techniques are essential to ensure reliable and reproducible radiomics-based models. For instance, the field strength in MRI (1.5T vs 3T) had a significant influence on a wide range of radiomics feature values, and images acquired with a distinct field strength in MRI should not be used interchangeably to build radiomics models (Ammari et al.).

### Hardware: New Devices, New Acquisition Protocols

Whatever imaging modality is considered, the improvement of patient management related to hardware evolutions perfectly illustrates the great contribution of technology in precision medicine. For example, preoperative Spectral CT imaging, which utilizes the energy spectrum curve and slope parameters, was beneficial in providing an objective approach to the preoperative staging of thymic epithelial tumors (Zhou et al.). Deep inspiration breath-hold reduced respiratory motion during radiotherapy allowing for more precise and accurate radiotherapy (Naumann et al.). An acquisition protocol incorporating deep inspiration breath-hold with optical surface-guided radiotherapy and image-guided radiotherapy was utilized to ensure reproducibility and accurate tumor localization in surface body radiotherapy (Naumann et al.). Finally, image-guided tissue biopsies using large gauge coaxial needles enabled multiple tissue biopsies in a single pass (Khan et al.). These high-yield samples could be beneficial in better understanding the molecular and genomic characteristics of tumors, ultimately assisting in the development of biomarkers of clinical and translational relevance (Khan et al.).

### Multimodal Hybrid Imaging

Multiparametric imaging offers unique opportunities to evaluate tumor characteristics at an advanced multidimensional imaging level. A literature review supported the utility of hybrid 18F-FDG PET/CT imaging modalities in the management of patients with muscle-invasive bladder carcinoma, suggesting that it may be used to guide precision medicine (Girard et al.). Additionally, PET, CT, and MRI used synergistically could provide complementary information with potentially beneficial clinical applications (Decazes et al.).

### Metabolic and Molecular Imaging

The pathogenesis of neoplastic tumors and their metabolic processes can be targeted through molecular imaging to better understand and characterize the tumor. PET imaging detection is based on the radionuclide labeling of molecular probes, providing almost unlimited opportunities to map numerous physiological or pathophysiological targeted processes. Numerous relevant molecular probes constitute a powerful arsenal to characterize several tumor biological processes *in vivo*. For example, isocitrate dehydrogenase enzyme (IDH) mutations is a known occurrence in gliomagenesis (Zhou et al.). A metabolic PET/CT-based nomogram model utilized easy-accessible imaging metrics and clinical features and was shown to provide predictive information for IDH mutational status in patients with gliomas (Zhou et al.). Another example is the use of fulvestrant, which is an estrogen receptor antagonist drug approved for postmenopausal women with HR-positive and HER2-negative metastatic breast cancer (Liu et al.). PET with dual tracers 16α-[18F]fluoro-17β-estradiol and 18F-FDG, was utilized as prognostic imaging biomarkers to predict the efficacy of fulvestrant therapy in patients with ER-positive metastatic breast cancer (Liu et al.).

## Guiding Initial Treatment Decision

### Precision Diagnosis: Grade, Stage, Genomics

The role of imaging in initial staging and treatment planning has been extensively demonstrated. Research combining initial imaging and artificial intelligence, including Radiomics, is a growing field that will help to better address some routine clinical challenges that every physician has to face.

As a radiologist, noninvasive image-based diagnosis can be challenging. As an example, differentiating solitary brain metastasis from glioblastoma multiforme can be difficult, and completely change patient’s management. To this end, a radiomics based classifier that evaluates the morphological differences on post-contrast 3DT1 weighted MRI was developed to distinguish between solitary brain metastasis and glioblastoma multiforme (de Causans et al.). Another example is the non-invasive detection of intrahepatic cholangiocarcinoma. Radiomics-based models may improve the diagnostic accuracy of intrahepatic cholangiocarcinoma (Xue et al.). In addition, Radiomics-based biomarkers can offer key insights into precision diagnostics, disease classification, prognostication, and therapeutic management in neuro-oncology (Shaikh et al.).

Lung cancer remains a leading cause of cancer-related deaths (El Ayachy et al.) despite recent therapeutic advances in the field such as immunotherapy. Radiomics was used to identify candidate biomarkers that could enhance our understanding of the microbiology of lung cancer (El Ayachy et al.). In addition, 18F-FDG PET/CT radiomics nomogram combined with clinicopathologic factors could be used to predict survival outcomes in patients with lung adenocarcinoma with an EGFR mutation (Yang et al.). This approach could be utilized to provide a more precise diagnosis and offer personalized treatment guidance for patients with EGFR mutations (Yang et al.).

Another example is the impact of imaging improvements in common cancers such as prostate cancer. Prostate lymph node dissection clinical nomograms have the potential to predict non-regional lymph node metastasis in prostate cancer patients (Jiao et al.). The use of clinical nomograms in conjunction with Ga-PSMA PET/CT could enhance the detection of distant lymph node metastasis and guide clinical decision-making (Jiao et al.).

### Baseline Prognostic and Predictive Imaging Biomarkers

Baseline imaging plays a key role in clinical care patient management. Indeed, it allows for assessing tumor burden, potential extension, and can also determine prognosis. The development of radiological or hybrid tools that combine several features will help to better target treatment in order to personalize therapies. For instance, a nomogram and risk stratification system in early-stage cervical cancer patients (stage IB1 and IB2) could have clinical utility in predicting progression-free survival (Xu et al.). SUV peak and HPV-16 were shown to independently impact disease progression (Xu et al.) Artificial Intelligence could also play a key role as prognosis tool. In (Wang et al.), CT radiomics offered an objective approach to predicting the risk of malignant gastrointestinal stromal tumors by using various machine-learning algorithms. Of note, machine-learning models require multicenter testing prior to their use in clinical decision-making (Wang et al.). Metabolic imaging plays a major role as a baseline investigation in clinical routine. It has also a dominant role in predicting response. In (Aide et al.), 18F-FDG PET metabolic heterogeneity of estrogen receptors was used to assist in noninvasively identifying patients with the worst event-free survival in breast cancer.

Baseline imaging is also used to guide treatment strategies in focal therapies such as radiation therapies. CT radiomics could be utilized to localize radioresistant sub-volumes in tumors, serving as a predictive biomarker in radiotherapy (Bogowicz et al.). This methodology could be employed to identify potential targets for dose intensification (Bogowicz et al.).

The use and application of new technologies in the medical field require significant scientific rigor and exact knowledge of limits and bias. This is all the more applicable in this era of artificial intelligence. For instance, it was demonstrated that prognostic radiomics signatures must control for confounding variables to avoid inaccurate prediction of survival outcomes in patients with clear cell renal cell carcinoma (Lu et al.).

## Assessing Tumor Sensitivity to Treatments

Immunotherapies have become part of the standard-of-care of a wide range of cancers. These therapies have improved clinical outcomes compared to other treatments, such as chemotherapy or targeted therapies. Unfortunately, only a fraction of patients experiences such therapeutic success. Therefore, the early identification of biomarkers in patients that are unlikely to benefit from immunotherapies is a crucial step in selecting appropriate candidates.

Chimeric antigen receptor (CAR) targeting CD19 antigen, a form of immunotherapy used to treat non-Hodgkin’s lymphoma, is frequently complicated by relapses (Vercellino et al.). This strongly suggests further evaluation of the use of medical imaging such as PET/CT for the monitoring of patient response and the optimization of medical care, including risk stratification (Vercellino et al.).

Another clinical drawback with immunotherapies relates to their safety. The incidence of severe immune-related adverse events is not negligible and can even lead to the death of the patient. In (Ederhy et al.) the focus was on cardiovascular toxicities, particularly myocarditis, under immune checkpoints inhibitors. This adverse effect should be recognized promptly due to the high fatality rate (30-50%) (Ederhy et al.). Medical imaging could play an important role in optimizing the management of immune-related myocarditis, including diagnosis, prognostication, treatment decision, and follow-up (Ederhy et al.).

Given the fact that immunotherapy is based on restoring tumor elimination by the immune system, novel patterns of response and progression have emerged, such as pseudoprogression, hyperprogression, abscopal effect, and durable response after treatment. Dissociated response, is an additional pattern defined as heterogeneous-responding lesions in the same patient. While dissociated response has historically been labeled as an unfavorable prognostic pattern, immunotherapy with immune checkpoint inhibitors has revealed that it could in fact be a favorable prognostic pattern (Humbert and Chardin).

New response criteria standardization has been developed, aiming to include these novel patterns of response and progression during the course of immunotherapy. In (Castello et al.), the development of the immune metabolic response criteria by 18FDG PET/CT, called “imPERCIST”, was shown to optimize the prediction of clinical benefit in immunotherapy regimens (Castello et al.). These criteria may be beneficial in evaluating the response to immunotherapy, and subsequently guiding clinical decision-making. However, it is important to note that a wide range of novel criteria have been designed for 18F-FDG PET/CT (i.e., PECRIT, PERCIMT, iPERCIST), and have not been prospectively validated. Hence, a large-scale multicenter validation should be performed prior to implementation in the daily routine.

A similar question regarding the response criteria is raised for patients with pancreatic neuroendocrine tumors. Specific criteria should be further developed since the current literature reveals a lack of standardization and comparable methodological approaches in the evaluation of pancreatic neuroendocrine tumors (Partouche et al.).

## Diagnosis and Management of COVID-19 in Cancer Patients

The pandemic of COVID-19 has impacted medical, economic, social, and environmental practices worldwide. During its peak, clinicians had to rethink the management of patients with a diagnosis of cancer. On the one hand, cancer patients are at increased risk of serious complications due to their comorbidities, therapy, and immune dysregulation. Therefore, one strategy was to delay medical imaging to avoid exposure and subsequent risk of infection. On the other hand, the role of imaging is critical for cancer management, hence delaying management can be detrimental to the patient’s prognosis. In this collection, several papers deciphered strategies to optimize the management of cancer patients while in treatment.

Patients requiring systemic therapies during the pandemic could be screened with reverse transcription-polymerase chain reaction (RT-PCR) prior to initiating therapy and rescreened at 15 days if positive. Those exhibiting respiratory symptoms or signs of acute inflammatory syndrome, could undergo a CT scan or CT angiogram, respectively, to guide clinical decision-making (Viansone et al.). Strategies were proposed so that radiation oncology departments could provide strategies to identify COVID-19 infection and ensure the optimization of patient care by the use of specific workflows, which include RT-PCR, CT scans, and social distancing (Sun et al.). Finally, strategies for screening and early detection of COVID-19 were proposed in patients with hematologic malignancies due to their immunosuppressive state, to mitigate the spread in this patient population (Assi et al.).

## Conclusion

In conclusion, this guest editorial shared proof of concepts results using disruptive technologies or concepts in the field of imaging-guided precision medicine in oncology. It further demonstrates that medical imaging is pivotal for a broad spectrum of indications. The next step forward will be the prospective validation of these findings in large multicenter prospective studies.

## Author Contributions

All authors listed have made a substantial, direct, and intellectual contribution to the work and approved it for publication.

## Conflict of Interest

The authors declare that the research was conducted in the absence of any commercial or financial relationships that could be construed as a potential conflict of interest.

## Publisher’s Note

All claims expressed in this article are solely those of the authors and do not necessarily represent those of their affiliated organizations, or those of the publisher, the editors and the reviewers. Any product that may be evaluated in this article, or claim that may be made by its manufacturer, is not guaranteed or endorsed by the publisher.

